# Method development and validation for the quantification of organic acids in microbial samples using anionic exchange solid-phase extraction and gas chromatography-mass spectrometry

**DOI:** 10.1007/s00216-020-02883-3

**Published:** 2020-09-24

**Authors:** Simone Heyen, Barbara M. Scholz-Böttcher, Ralf Rabus, Heinz Wilkes

**Affiliations:** Institute for Chemistry and Biology of the Marine Environment (ICBM), Carl von Ossietzky University of Oldenburg, P.O. Box 2503, 26111 Oldenburg, Germany

**Keywords:** Calibration, Recovery, Stability, Limit of detection, Limit of quantification, Statistics

## Abstract

Organic acids play a key role in central metabolic functions of organisms, are crucial for understanding regulatory processes and are ubiquitous inside the cell. Therefore, quantification of these compounds provides a valuable approach for studying dynamics of metabolic processes, in particular when the organism faces changing environmental conditions. However, the extraction and analysis of organic acids can be challenging and validated methods available in this field are limited. In this study, we developed a method for the extraction and quantification of organic acids from microbial samples based on solid-phase extraction on a strong anionic exchange cartridge and gas chromatographic-mass spectrometric analysis. Full method validation was conducted to determine quality parameters of the new method. Recoveries for 12 of the 15 aromatic and aliphatic acids were between 100 and 111% and detection limits between 3 and 272 ng/mL. The ranges for the regression coefficients and process standard deviations for these compound classes were 0.9874–0.9994 and 0.04–0.69 μg/mL, respectively. Limitations were encountered when targeting aliphatic acids with hydroxy, oxo or enol ester functions. Finally, we demonstrated the applicability of the method on cell extracts of the bacterium *Escherichia coli* and the dinoflagellate *Prorocentrum minimum*.

**Electronic supplementary material:**

The online version of this article (10.1007/s00216-020-02883-3) contains supplementary material, which is available to authorized users.

**Figure:**
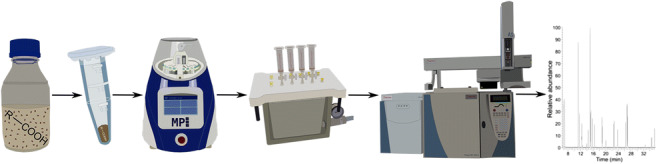
Graphical abstract

## Introduction

Metabolomics denotes the investigation of the metabolite composition in cells, tissues or organisms and belongs to the “-omics” disciplines—genomics, proteomics and metabolomics—converging in systems biology [1]. In the last years, growing interest has shed light on this emerging field especially in biotechnology and clinical research [2, 3]. With an increasing number of publications relating to metabolomics, more analytical approaches have advanced, including gas or liquid chromatography coupled to mass spectrometry (GC-MS, LC-MS) and nuclear magnetic resonance (NMR) spectroscopy [4, 5]. Besides an overall examination of the metabolome [6], two separate fields have developed—the analysis of the metabolic composition outside of the cell, also called the exometabolome or metabolic footprinting [7] and the analysis of the metabolism inside of the cell, termed endometabolome or metabolic fingerprinting [8, 9]. Methods used for the extraction of intracellular metabolites mostly include organic solvents or a mixture of different solvents after quenching of the cells to halt metabolic activities [10]. However, depending on the choice of the extraction method, the gained data and therefore the interpretation is influenced [11]. For example, by comparing four extraction methods for intracellular metabolites, Duportet et al. found that a significant proportion of the identified metabolites appear to be method-specific [12]. Therefore, detailed insight into certain functions can only be accessed if metabolic key compounds are targeted. Members of the tricarboxylic acid cycle (TCA cycle) as well as other organic acids (OAs) play a major role in understanding metabolic processes, as OAs are ubiquitous inside cells and the TCA cycle is not only the tail end of the catabolism but also provides precursors for anabolism [13, 14].

For the extraction and analysis of OAs, several protocols and procedures have been developed. Markuszewski et al. used capillary electrophoresis with indirect UV detection for the analysis of metabolites from the TCA cycle in *Bacillus subtilis* [15]. Kombu et al. described a method for the extraction of intermediates of the TCA cycle with chloroform/methanol or 5% acetic acid in methanol/water followed by GC-MS analysis [16]. An internal standard was used to correct for losses during the procedure. Mamer et al. developed a stable isotope dilution method for tissue grown on culture plates [17]. In all of these studies, extracts were measured directly after disruption of the cells, leading to the possibility of matrix effects causing ion suppression during measurement, which induces reduced sensitivity as well as increased detection and quantification limits [18]. One possibility to improve the chromatographic resolution of cell extracts when targeting specific compounds is the application of an additional extraction step to remove disturbing matrix compounds, which can influence the measurement [19].

Solid-phase extractions (SPEs) are widely used in sample preparation and new methods and materials were developed over the last decades [20]. When targeting OAs, anionic exchange sorbents are often chosen [21–23]. However, most methods utilizing SPE to gain quantitative data on OAs focus on food and clinical research [23, 24]. In this, concentrations of OAs are considerably higher and the validation applied in these studies cannot be transferred onto microorganisms by implication. Consequently, method development and validation for microorganisms needs to focus on establishing satisfying recoveries as well as very low detection and quantification limits to detect analytes present in traces only. Besides the extraction and clean-up procedure, the method used for the determination of the analytes has an additional influence on the sensitivity. GC-MS offers the advantage of minimizing problems related to ion suppression of coeluting compounds as well as eliminating discrimination of compounds due to response differences and is, besides LC-MS, one of the main application for endometabolome analyses [25, 26]. Factors affecting the quantitative analysis of metabolites using GC-MS have been reviewed by Koek et al. [27]. They emphasized the importance of using a set of standard compounds as well as a proper method validation. The authors also documented that only a limited number of studies gaining validated quantitative data in microbial samples exist. Before starting routine applications, proper method validation should be performed as biases may alter the results when neglected [28]. Otherwise frequently encountered problems are related to the derivatization, inevitable for GC-MS analysis of OAs, as instability may occur and reaction kinetics change based on the functional groups of the derivatized compounds [29, 30].

In this study, we developed a method for the extraction of OAs from cell extracts with an anionic exchange solid-phase extraction cartridge and used gas chromatography-mass spectrometry for the determination of the target compounds. We targeted 24 OAs, which included intermediates of the TCA cycle as well as OAs known to occur in the metabolic pathway of microorganisms studied in our laboratory and may be interesting for future quantitative studies [31]. The linearity of the method, storage stability and stability of the derivatized OAs, limit of detection and quantification as well as the recoveries were evaluated as validation parameters before applying the method to extracted cells of a bacterium, *Escherichia coli*, and a dinoflagellate, *Prorocentrum minimum*.

## Experimental

### Chemicals

Lactic acid (sodium salt), pyruvic acid (sodium salt), malic acid, maleic acid, glutaric acid and ammonium hydroxide solution (25%) were purchased from Fluka (Neu-Ulm, Germany). Malonic acid (sodium salt), succinic acid, maleic acid and formic acid were bought from Merck (Darmstadt, Germany). Fumaric acid, α-ketoglutaric acid and citric acid were from Carl Roth GmbH + Co. KG (Karlsruhe, Germany). Phenylacetic acid and hypoxanthin were purchased from Acros Organics (NJ, USA). Glycolic acid, phosphoenolpyruvic acid (monopotassium salt), glutaric acid, pimelic acid, methylmalonic acid, methylsuccinic acid, oxaloacetic acid, isocitric acid (trisodium salt), benzoic acid, hydrocinnamic acid, cinnamic acid, *p*-hydroxybenzoic acid, benzylsuccinic acid, L-4-thiazolidinecarboxylic acid, L-pyroglutamic acid, adenine, DL-glutamic acid, DL-phenylalanine, DL-methionine, DL-aspartic acid, *trans*-(β,2,3,4,5,6-^2^H_6_)cinnamic acid, (2,3,4,5,6-^2^H_5_)benzoic acid and (2,2,3,3-^2^H_4_)succinic acid were bought from Sigma-Aldrich (Steinheim, Germany). Adipic acid was from Riedel-de-Haën (Seelze, Germany). 5α-Androstan-17-one was purchased from SERVA Electrophoresis GmbH (Heidelberg, Germany). *N*-Methyl-*N*-(trimethylsilyl)trifluoroacetamide (MSTFA) was bought from CS Chromatography Service GmbH (Langerwehe, Germany) and methanol from Biosolve Chimie SARL (Dieuze, France). Dichloromethane from various suppliers was freshly distilled and checked for impurities via gas chromatography.

Mixed stock solutions of 1 mg/mL (0.5 mg/mL for (2,3,4,5,6-^2^H_5_)benzoic acid and 0.25 mg/mL (2,2,3,3-^2^H_4_)succinic acid were prepared in double distilled water with a pH adjusted to 9 using an ammonium hydroxide solution.

### Cultivation of microorganisms

The bacterium *Escherichia coli* K12 (DSM 18039) was obtained from the German Collection of Microorganisms and Cell Cultures (DSMZ; Braunschweig, Germany). *E. coli* K12 stock cultures were revived in LB (Luria–Bertani) medium (DSMZ medium no. 381), followed by adaptation over several passages to defined mineral M9 medium (DSMZ medium no. 382) containing glucose (6 mM) as sole source of organic carbon and energy as well as 1 mL/L of a trace element mixture [32]. Growth of cultures was monitored by measuring the optical density (OD) at 600 nm (UVmini-1240; Shimadzu, Duisburg, Germany). Main cultures were performed in triplicates using 1 L Erlenmeyer flasks (containing 250 mL medium, inoculated to OD_600_ 0.02) and by incubating at 37 °C on a rotary shaker (100 rpm). Cultures were harvested at ½ OD_max_ (0.7), including the following steps: centrifugation of 250 mL culture broth (14,000*g*, 15 min, 4 °C), resuspending the cell pellets in 200 mL Tris/HCl buffer (100 mM Tris, 5 mM MgCl_2_ × 6 H_2_O, adjusted to pH 7.5), anew centrifugation (20,000*g*, 15 min, 4 °C) and resuspending in 0.8 mL Tris/HCl buffer, shock freezing the cell pellets in liquid N_2_ and storage at − 80 °C until further analyses.

The dinoflagellate *Prorocentrum minimum* CCMP 1329 was obtained from the Bigelow National Center for Marine Algae and Microbiota (East Boothbay, ME, USA) via the Wagner-Döbler lab (Technische Universität Braunschweig, Germany). *P. minimum* was cultivated in defined mineral L1-Si medium adjusted to pH 8.3 (for details see: ncma.bigelow.org/ccmp1329). A special incubation cabinet equipped with two-sided, thermally insulated lighting (P530; Rubarth Apparate GmbH, Laatzen, Germany) was used, applying the following settings: 12 h light/12 h dark cycle, 10% light, 50% air circulation and 20 °C. Cultivation was carried out in 1 L Erlenmeyer flasks (containing 100 mL medium) without shaking. Growth of cultures was monitored by measuring the OD at 440 nm. For harvesting, actively growing cells of three replicate cultures were pooled, followed by three rounds of gentle centrifugation (2000*g*, 10 min, 4 °C) and washing with Tris/HCl buffer (see above), with the buffer volume decreasing from 100 mL via 5 mL to 1 mL. The resultant cell pellets were shock frozen in liquid N_2_ and stored at − 80 °C until further analyses.

### Cell extraction

Cells from *E. coli* and *P. minimum* cultivation experiments were disrupted as follows. Cell pellets were transferred into pre-weighted Cryotubes filled with 0.5 g of 0.1 mm glass beads and 1 g of 0.7 mm zirconia beads. One millilitre of methanol was added before treating the cells in the bead beater homogenizer (Fast Prep-24 5G; MP Biomedicals, Santa Ana, CA, USA) at 6 m/s for 30 s. This procedure was repeated twice with a cooling phase of 2 min in between cycles to prevent decomposition of metabolites. Afterwards, the samples were centrifuged at 4 °C and 12,000 rpm for 10 min (Sigma 4K10, Osterode am Harz, Germany). While the methanol was transferred into a 4 mL glass vial and evaporated, the centrifugation was repeated twice after resuspending the cells with 1 mL double-distilled water adjusted to pH 9 with a 5% ammonium hydroxide solution. The pooled extract was treated in an ultrasonic bath before adding the internal standard (IS) and submitting the sample to solid-phase extraction.

### Solid-phase extraction

For solid-phase extraction, 150 mg Oasis MAX cartridges were used (Waters, Eschborn, Germany). Prior to extraction, samples were basified with a 5% ammonium hydroxide solution to pH 9. After conditioning and equilibrating the cartridges with 4 mL each of methanol and water, the pH-adjusted sample was applied with a flow rate below 1 mL/min. Four millilitres of 5% NH_4_OH were used as washing solution and the cartridge was dried by applying a vacuum for 30 min. Carboxylic acids were eluted with 2 mL 5% formic acid in methanol twice.

### Derivatization

For derivatization, we chose silylation, which has already been established as valid methodology for OAs by Koek et al., and to ensure that the smallest OAs were accessible with our GC-MS method [26]. One hundred microlitres of each sample were evaporated at 50 °C under a nitrogen stream to complete dryness and resolved in 50 μL dichloromethane and 40 μL MSTFA. Samples were silylated at 70 °C for 2 h. After the sample had cooled down to ambient temperature, 10 μL of the injection standard containing 5α-androstan-17-one (10 μg/mL) was added before GC-MS analysis. As some signals exceeded the limits of our calibration range, biological samples were additionally measured using only 25 μL sample volume for derivatization.

### GC-MS analysis

Gas chromatographic-mass spectrometric analyses of silylated standards and extracts were performed on a Trace GC Ultra gas chromatograph coupled to a TriPlus autosampler and an ISQ QD mass spectrometer operated with the Xcalibur software, version 4.1.31.4 (all Thermo Scientific, Bremen, Germany). The gas chromatograph was equipped with a PTV injector in splitless mode and an Agilent J&W DB-5 capillary column with a length of 30 m, an inner diameter of 0.25 mm and a film thickness of 0.25 μm. The tray of the autosampler was cooled at 15 °C. One microlitre of sample solution was injected into the system. The oven temperature program started at 70 °C, was kept for 2 min and then raised to 200 °C with a heating rate of 3 K/min. A second ramp followed with a heating rate of 20 K/min, until a final temperature of 320 °C was reached, which was held for 10 min. The total runtime was 61.33 min. Helium was used as carrier gas. The ionization mode for the mass spectrometer was electron impact at 70 eV. The transfer line temperature was 280 °C and the ion source temperature was 220 °C. Full-scan mass spectra were recorded between *m*/*z* 50 and 650 at a scan time of 0.2 s. For peak integration, a set of characteristic and selective fragments (Table 1) was chosen for each acid in order to enhance sensitivity of the method.

**Table 1:** Performance indicators as well as MS fragments used for quantification

Organic acid	Range (μg/mL)	*R*^2^	Process standard deviation (μg/mL)	Recovery (%)	Limit of detection DIN/SNR (ng/mL)	Limit of quantification DIN/SNR (ng/mL)	RSD of derivatized samples at 4 or 17.5 μg/mL (%)	RSD of derivatized samples at 0.2 or 2 μg/mL (%)	Molecular ion (*m*/*z*)	Fragments added up for quantification (*m*/*z*)
*Aromatic acids (Ar)*										
Benzoic acid	0.01–5	0.9994	0.04	100	30/10	110/10	6	9	194	194, 179, 135, 105, 77, 51
Phenylacetic acid	0.01–5	0.9980	0.08	105	15/10	50/50	6	8	208	200, 193, 164, 91
Hydrocinnamic acid	0.01–5	0.9991	0.05	100	8/10	25/50	5	6	222	222, 207, 104
Cinnamic acid	0.01–5	0.9989	0.06	100	4/10	15/10	4	5	220	220, 205, 161, 131, 103
*p*-Hydroxybenzoic acid	0.01–5	0.9978	0.08	111	33/10	149/10	5	4	282	282, 267, 223, 193, 151, 126
Benzylsuccinic acid	0.01–5	0.9869	0.20	110	74/10	594/50	2	3	352	352, 337, 321, 293, 275, 262, 247, 234, 221, 205, 190, 175, 131, 91
*Aliphatic dicarboxylic acids (Al)*										
Malonic acid	0.5–20	0.9907	0.69	54	121/500	344/500	4	4	248	248, 233, 133
Methylmalonic acid	0.05–5	0.9983	0.08	27	21/50	72/50	6	6	262	262, 247, 218
Maleic acid	0.1–20	0.9967	0.41	0.3	272/100	861/500	6	6	260	245, 133, 115, 83
Succinic acid	0.01–5	0.9994	0.04	109	22/10	75/10	6	8	262	247, 129
Methylsuccinic acid	0.01–5	0.9975	0.09	103	19/10	62/50	6	7	276	262, 232, 217, 186
Fumaric acid	0.01–5	0.9940	0.14	100	3/10	5/10	6	8	260	245, 83
Glutaric acid	0.01–5	0.9950	0.13	104	15/10	53/50	5	7	276	261, 233, 204, 186, 158, 97
Adipic acid	0.01–5	0.9931	0.15	104	30/10	110/10	5	6	290	290, 275, 204, 185, 172, 141
Pimelic acid	0.01–5	0.9874	0.20	101	23/10	105/50	4	5	304	304, 289, 217, 186, 173, 155
*Aliphatic acids with a hydroxy group (OH)*										
Lactic acid	0.01–5	0.8314	0.81	27	115/10	1367/10	7	32	234	219, 191
Glycolic acid	0.01–5	0.9760	0.28	5.5	41/10	175/10	6	10	220	205, 177, 161, 133
Malic acid	0.05–5	0.8930	0.63	151	98/50	1224/50	5	5	350	335, 307, 265, 245, 233, 189, 175, 133, 101
Citric acid	0.05–5	0.7799	0.97	125	45/50	172/100	2	3	480	465, 375, 363, 347, 273, 257, 183
Isocitric acid	0.05–5	0.8692	0.71	161	38/50	121/100	3	3	480	465, 375, 363, 347, 319, 273, 245
*Aliphatic acids with an oxo or enol ester group (O)*										
Phosphoenolpyruvic acid	1–20	0.9691	1.20	0	1477/1000	5899/1000	2	12	384	384, 369, 299, 243, 217, 211, 133
Pyruvic acid	0.01–5	0.8667	0.71	19	50/10	255/10	7	16	232	217, 189
α-Ketoglutaric acid	0.5–20	0.9387	1.81	273	437/500	1594/500	10	7	362	362, 347, 318, 291, 157, 113
Oxaloacetic acid	3–20	0.8575	2.58	16	5577/3000	20,564/6000	14	–	348	333, 305, 231, 221, 171, 133

### Validation

Before applying the method to biological samples, several performance indicators needed to be evaluated. For this purpose, experiments to access the calibration range, limits of detection and quantification, stability as well as recovery of the acids were performed before applying the method to cell extracts of *E. coli* and *P. minimum*.

#### Calibration

Calibration was performed in the range of 0.01–5 μg/mL and 0.1–20 μg/mL for compounds with a limited response in the GC-MS system. We chose eight calibration points distributed over the concentration range with six replicates for each concentration, leading to a total of 48 measurements per calibration. Outliers within calibration points were determined using the Grubbs test, testing the minimum and the maximum value of each concentration against the mean value of all replicates [33, 34]. If the calculated result was higher than the tabulated control value for the Grubbs test at a confidence level of 95%, the data point was considered a significant outlier and removed from the calibration. The square of the correlation coefficient as well as the process standard deviation were considered as qualitative markers of the calibration. Lastly, we calculated 95% confidence and prediction bands [35] of all calibrations.

#### Limits of detection and quantification

For the calculation of the detection and quantification limits (LOD and LOQ), we prepared 19 samples between 1 and 100 ng/mL (10 and 1000 ng/mL for low response acids) to have sufficient data points close to the assessed detection limit for all acids. At least five of these samples were used to calculate the limits according to DIN 32645 with the lowest concentration near and the highest concentration not more than ten times the estimated detection limit [36].

#### Storage stability

To test the stability of the underivatized solutions under different conditions, the stock solutions of the analytes were diluted to a high (4 and 17.5 μg/mL for high and low response OAs, respectively) and a low (0.2 and 2 μg/mL for high and low response OAs, respectively) concentration relative to the chosen calibration range. Five of these samples each were measured directly after preparing the solutions and on the same day the stock solutions were made. Seventy-two aliquots of both concentrations were stored either at room temperature, 4 °C or −18 °C and measured over a period of 3 weeks in triplicates. Additionally, the stock solution, which was stored at −18 °C, was diluted to the higher concentration level and analysed in triplicates on the same days as the other stability samples.

#### Stability of derivatized organic acids

Assessing the stability of processed and derivatized samples is necessary to detect any decomposition occurring during residence time in the autosampler as measurement might start a couple of hours after derivatization when a longer sequence is prepared. To investigate whether silylated samples are affected, solutions of analytes were prepared in the same two concentrations as were used for the storage stability tests. A 2-mL sample was derivatized and divided into 15 aliquots, which were measured in sequence. The relative standard deviation (RSD) was then used to examine any significant variances in the area ratios in relation to the injection standard.

#### Recovery

The extraction efficiency indicates if the analyte is fully transferred from the matrix into the primary extract. It is determined by performing two correlated experiments. In the first one, blank matrix is spiked with the analyte prior to and the IS after extraction. In the second experiment, blank matrix is extracted and afterwards spiked with the same concentrations of analyte and IS. The percentage of the first analyte/IS ratio of the ratio calculated for the second experiment displays the extraction efficiency. As this can depend on the concentration of the analyte, we performed this experiment at six concentrations distributed over the calibration range in duplicates. Recoveries were determined as the slope of the regression curve given by plotting the area ratio of the samples with IS added after extraction against the area ratio of the samples with IS added before SPE. To access the recovery of the internal standard, blank matrixes were spiked with IS prior to solid-phase extraction and compared to the samples spiked with IS after SPE.

#### Further statistical evaluation

For proper statistical evaluation, multiple tests are recommended, which were applied to the present dataset. Gaussian distribution within concentration levels of the calibration was examined applying the Shapiro–Wilk and the David tests [37, 38]. As for standard applications, confidence levels of 95% for the Shapiro–Wilk and 90% for the David test were chosen. The Mandel test was applied to test the linearity of the calibration. This test as well as a test for homogeneity of variances was applied as described in DIN 38402 part 51 [39]. Additionally, variances across the calibration range were evaluated according to Cochran’s *C* test with 95% confidence [40]. The results and discussion of these calculations can be found in the Electronic Supplementary Material (ESM).

### Application

We applied the new method to three biological replicates each of *E. coli* and *P. minimum*. Each SPE extract was measured three times. Concentrations of acids in cell extracts were calculated by assigning the ratio of the area of the analyte and the area of the internal standard to the corresponding values in the calibration curve. Confidence intervals for the concentrations provided in this way were defined according to DIN 38402 part 51 [39].

## Results and discussion

### Method development

**Fig. 1:**
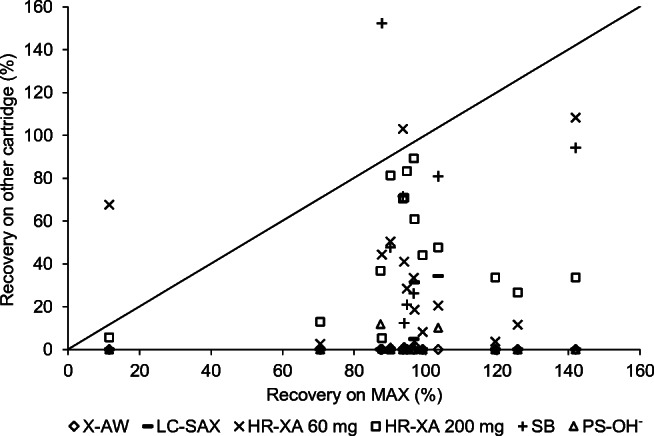
Comparison of the recoveries of a test mixture on six cartridges to the extraction efficiency of the Oasis MAX cartridge. The diagonal line indicates equal recovery on both cartridges. Everything above the line was better retained on the cartridge in comparison, while everything below the line was better retained on the Oasis MAX cartridge

In contrast to untargeted extraction processes, SPE specifically intended for a certain compound class offers the possibility to detect and quantify compounds which may otherwise be overlooked when analysing a complex sample mixture if the concentration of the analyte is low. In order to determine the best method to extract OAs, different procedures were evaluated. First, we tried to apply liquid–liquid extraction (LLE), as this is a method routinely used in our lab for metabolite extraction. Tests performed with dichloromethane or diethyl ether and ethyl acetate as solvents did not show satisfactory results, as just a few acids from the test mixture could be retrieved (data not shown). Additionally, SPE is faster, consumes less solvent and does not lead to the formation of emulsions as it can be the case with LLE, which will be hard to disrupt and may lead to loss of analytes when working with biological samples. Besides the Oasis MAX cartridges, we tested six other anionic exchange cartridges containing material potentially suitable for the extraction of OAs. The cartridges tested were Chromabond HR-XA, PS-OH^−^ and SB, Supelclean LC-SAX as well as Phenomenex Strata X-AW. We compared the recoveries of some selected acids achieved with the MAX cartridge to the results of the other cartridges when applying standard protocols as given by manufacturers (Fig. 1 and ESM Table S1).

**Fig. 2:**
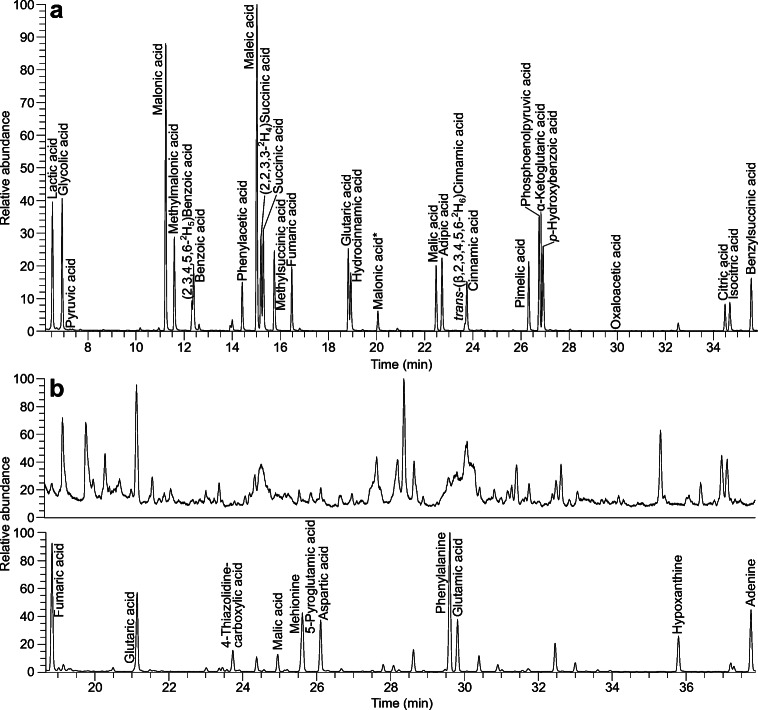
Sections of interest of the **a** total ion chromatogram (TIC) of the standard mixture and **b** TIC of a silylated *E. coli* extract after bead beating (upper chromatogram) and OAs found in extracts after additional solid-phase extraction on Oasis MAX cartridges (lower chromatogram). Compounds not part of this study were identified by comparison with authentic standards. *3TMS derivative, which was not considered for quantitative analysis, as peak ratios remained constant over the period of the experiments (ratio peak height 2TMS/3TMS = 13.9, SD = 1.8, *n* = 25)

As the recovery was best with the Oasis MAX cartridges, we decided to use this cartridge and to optimize the SPE to further enhance the recovery. To test for losses during the extraction procedure, we checked the sample volume which passed through the cartridge, as well as the fraction captured after methanol was applied, which elutes the neutral fraction of a sample, for residues of the analytes. Of the 17 acids present in the test mixture, traces of five of them could be found in the part of the sample which passed through the cartridge with concentrations below 3% of the reference sample. Contents in the neutral fraction of the SPE were not consistent but existed. Therefore, we omitted the washing/elution step with methanol from our procedure and added a drying step under vacuum to remove the water left on the cartridge. We also tested the coupling of two cartridges on top of each other or performing the extraction in a batch process, which however both lead to a reduced recovery of OAs. As we assumed that loss of analytes occurs due to a strong binding to the cartridge material, we examined if the inclusion of a second extraction step and an increase of the amount of formic acid to 10% would enhance the recovery. However, analytes were not found in any of these additional fractions obtained. According to the supplier, the recommended pH of the sample before submitting it to solid-phase extraction on Oasis MAX cartridges is 10. For optimal performance, pH should at least be two units above the p*K*_a_ of the analyte. Most of the acids considered in this study have a p*K*_a_ of around 4. We tested the recovery with pH values between 7.8 and 10 to assure applicability for acids with higher p*K*_a_ for future extension of the analytical range. A pH of 9 yielded better recoveries than the experiments done at a pH of 10, while it was still high enough to ensure that all the analytes were present in their dissociated form (ESM Fig. S1). We also optimized the temperature program of the GC oven to enable best possible peak shapes and separation as dichloromethane and MSTFA differ significantly in their boiling points (39.6 versus 131 °C). In conclusion, the starting temperature should not be too high to prevent peak fronting for the low-boiling compounds while also being high enough to prevent peak fronting for the later eluting analytes. We tested different temperatures between 60 and 80 °C and found that 70 °C was the preferred temperature to ensure these criteria (ESM Fig. S2). The chromatogram achieved with the final settings including all analytes accessed in this study and the improvement achieved by using SPE in addition to bead beating can be seen in Fig. 2.

### Validation

For the presentation of the following sections, acids were sorted into four different categories according to their chemical properties as listed in Table 1.

#### Calibration

**Fig. 3:**
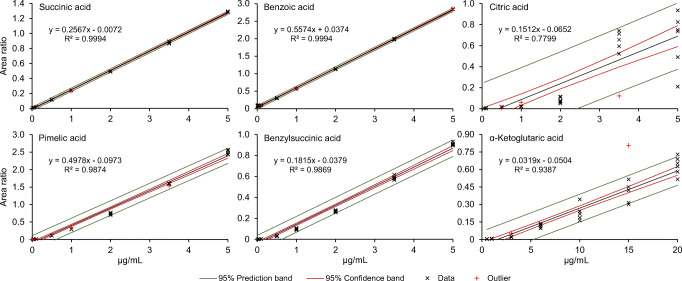
Exemplary depiction of calibration parameters for six OAs. Each graph shows area ratios of the acids with the corresponding internal standard for all measured concentrations and replications. Outliers eliminated via the Grubbs test are marked with a red cross. The regression lines in black including the formula and the squared correlation coefficients are displayed as well as the 95% confidence (red) and prediction (green) bands. Graphs for the other acids can be found in ESM Fig. S3

Calibration levels were chosen to be 0.01, 0.05, 0.1, 0.5, 1, 2, 3.5 and 5 μg/mL (0.1, 0.5, 1, 3, 6, 10, 15 and 20 μg/mL for acids with a low response factor). However, not all acids could be detected in the highly diluted samples. The ranges of the calibration for the individual acids as well as the squared regression coefficients and the process standard deviations for each calibration can be found in Table 1. The Grubbs test led to an elimination of outliers within calibration levels. Out of 1086 data points, 55 were classified as outliers, with a maximum of one outlier per concentration and between zero and five eliminated data points in total for one compound. The best results with *R*^2^ over 0.98 and the lowest process standard deviations were achieved for aromatic acids and aliphatic dicarboxylic acids without additional functional groups like hydroxy and oxo groups. In contrast, functionalized aliphatic acids showed poorer regression coefficients and higher variations in the process standard deviations, which is also reflected by broader confidence and prediction intervals for these compounds as depicted in Fig. 3.

#### Limits of detection and quantification

Of the samples measured to calculate LODs and LOQs, between five and ten were used to obtain these limits. The results acquired by applying the formulas given in DIN 32645 are shown in Table 1 [36]. For some acids, the concentrations of these samples were not high enough to have at least five data points for the evaluation. In the case of citric and phosphoenolpyruvic acid, two and three, respectively, additional concentration levels were used from the calibration, which still fulfilled the requirement of not being higher than ten times the estimated detection limit. LOD and LOQ for oxaloacetic acid were calculated using the calibration only because it could not be detected in any of the samples produced especially for LOD and LOQ. The detection limits were below 0.1 μg/mL for 75% of the acids. Acids with a reduced response factor constituted exceptions together with lactic acid. LOQs ranged from 5 ng/mL for formic acid to over 20 μg/mL for oxaloacetic acid, which would therefore be excluded from quantification with the calibration produced in this study. However, the signal-to-noise ratio (SNR), which is also an indicator for detection and quantification limits, was above 3 for the first calibration point for all analytes and above 10 for the first or the second concentration, indicating lower LODs and LOQs when determining the limits in this way, which may be a reason why this method is applied more often than the calculation according to the DIN norm.

#### Storage stability

**Fig. 4:**
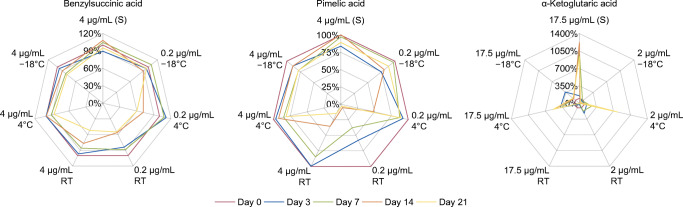
Examples of the storage stability over 21 days depicted as net graphs. Mean values of relative intensities measured on each day are given as percentage of day 0. (S) Diluted stock solution stored at *−* 18 °C. Graphs for the other acids can be found in ESM Fig. S4

As expected, acids were least stable at room temperature, although some acids did not start to decompose significantly before day 14 when stored in a higher concentration. Stability was best for aromatic acids, followed by dicarboxylic aliphatic acids without additional functional groups. Intensities of functionalized aliphatic acids with hydroxy, oxo or enol ester groups decreased the most or even enhanced extensively especially in the diluted stock solutions. This is in accordance with Christou et al., who documented a limited stability for glycolic and 3-hydroxypropionic acid stored in methanol at − 24 °C for 22 days, whereas unfunctionalized dicarboxylic acids remained stable [41]. α-Keto acids like oxaloacetic, pyruvic or α-ketoglutaric acid may decarboxylate spontaneously resulting in a limited stability of these acids. In cultivation experiments, this decarboxylation can additionally be facilitated by the addition of inorganic salts necessary for the growth of the organism [42, 43]. However, the extreme increase in intensities throughout the experiment for α-keto acids as well as variations within triplicate measurements cannot be explained at this moment. Some acids also showed enhancements up to around 200% in the samples with lower initial concentrations stored at − 18 °C or 4 °C, which may be caused by an incomplete dissolution of the acids on the day the stock solutions were prepared. Samples with higher concentration stored at − 18 °C or 4 °C generally had a better stability and partially were not affected during the complete analysis period. There was no trend in decomposition over time valid for all acids. While some acids seemed to be mostly unaffected for the investigated time period, other acids showed a considerable decrease in response after 3 days already. Best stability was observed for samples stored at − 18 °C. When assuming low sample concentrations, a fast preparation is recommended to reduce problems arising from instable compounds especially when they are highly functionalized. Examples for the stability over time for different storage conditions are given in Fig. 4.

#### Stability of derivatized organic acids

Stability for the samples left in the autosampler for 15 h varied between 2 and 14% RSD for the high concentration and between 3 and 32% RSD for the samples prepared at a lower concentration (Table 1). In the high concentration samples, RSD was at or below 7% except for α-ketoglutaric and oxaloacetic acid, which both contain an oxo group. For the lower concentration, RSD was at or below 10% except for phosphoenolpyruvic, pyruvic and lactic acid, which contain enol ester, oxo and hydroxy functions, respectively. The higher deviations for these acids may derive from the sensitivity of derivatives with trimethylsilyl esters as well as ethers to the presence of water even at trace levels. Variations in peak areas for functionalized aliphatic acids rather resulted from individual derivative properties, which are different for every acid, hard to predict and not necessarily time dependent. In conclusion, longer measurement sequences are possible, which is in agreement with previous studies [41, 44, 45].

#### Recovery

For aromatic and most dicarboxylic aliphatic acids without further functional groups, recovery was satisfactory with 100 to 111% as shown in Table 1. Phosphoenolpyruvic acid was the only compound which could not be retrieved at all. Recovery for the other aliphatic acids with a hydroxy or oxo function was either below 27% or above 125%. Interestingly, fumaric acid, the *trans* isomer of butenedioic acid, exhibited a very good recovery with 100%, whereas for the *cis* isomer, maleic acid, recovery was 0.3% even though they showed comparable signal responses. For the internal standard, recovery was 97% for *trans*-(β,2,3,4,5,6-^2^H_6_)cinnamic acid, 92% for (2,3,4,5,6-^2^H_5_)benzoic acid and 109% for (2,2,3,3-^2^H_4_)succinic acid, making all of them suitable for the application.

### Principal component analysis of performance indicators

**Fig. 5:**
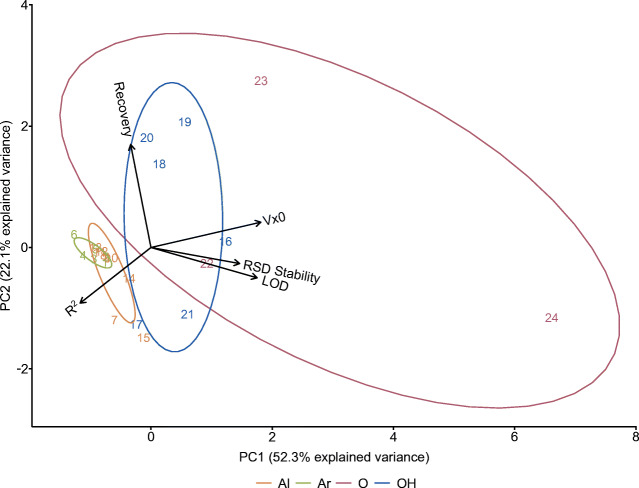
Principal component analysis of all OAs. Parameters used to calculate variances were the square of the regression coefficient of the calibration *R*^2^, the recoveries, process standard deviation Vx0, limit of detection LOD and the RSD of the derivatized samples at the higher concentration level (RSD stability). Ellipses were drawn around each compound class defined as described in Table 1. Ar: 1, benzoic acid; 2, phenylacetic acid; 3, hydrocinnamic acid; 4, cinnamic acid; 5, *p*-hydroxybenzoic acid; 6, benzylsuccinic acid; Al: 7, methylmalonic acid; 8, succinic acid; 9, methylsuccinic acid; 10, fumaric acid; 11, glutaric acid; 12, adipic acid; 13, pimelic acid; 14, malonic acid; 15, maleic acid; OH: 16, lactic acid; 17, glycolic acid; 18, malic acid; 19, citric acid; 20, isocitric acid; O: 21, phosphoenolpyruvic acid; 22, pyruvic acid; 23, α-ketoglutaric acid; 24, oxaloacetic acid

Considering the possible extension of the method to further target compounds, PCA analysis is a good tool to compare the performance indicators determined for our validation and to allow predictions of the behaviour of other analytes by investigating clustering of compounds or compound classes. As depicted in Fig. 5, aromatic acids and dicarboxylic aliphatic acids without further functional groups are highly correlated and cluster together, indicating a similar behaviour. In contrast, hydroxy-, oxo- and enol ester-group-containing aliphatic acids are spread across the axes. They show high variations in comparison to the other groups as well as within the class associated with the same functional group, as they are being influenced by various parameters. This complicates a prediction of their behaviour when transferring our approach to other functionalized OAs.

### Comparison with other studies applying SPE

**Fig. 6:**
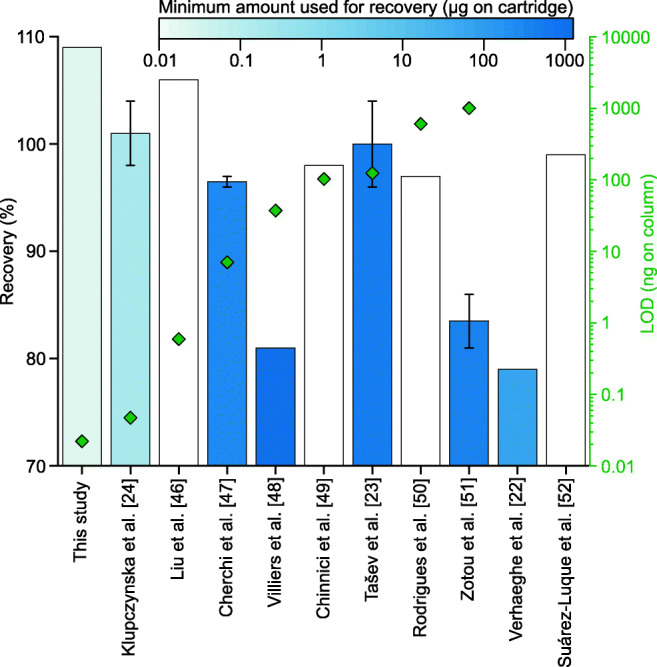
Recovery (bars), minimum amount used for the calculation of the recovery (colour of the bars) and calculated limits of detection (green rhombi) for succinic acid as evaluated in this study as well as in the studies used for comparison. White bars illustrate that no data for the amount of acid used for the determination of the recovery was provided as well as missing rhombi indicate that no LOD was given in the respective study. Error bars represent the lower and upper limits of the determined recovery. The lowest concentration used in the studies to calculate the recovery was considered in this depiction

To compare our results with previously published data, we examined ten publications, which all applied solid-phase extraction for the analysis of OAs and validated the recoveries and, where stated, the detection and quantification limits. We recalculated the given concentrations to the absolute amount of acid applied either to the SPE cartridge for the evaluation of the recovery or to the chromatographic column for the determination of the detection limit based on the data and information given in the papers. As it was the only OA accessed in all studies, Fig. 6 shows the results for the recovery including the recalculated amount used for the validation as well as the LODs of succinic acid. The full information on all OAs with the respective matrices, analytes covered, applied SPE technique and material, derivatization method (where applicable), recovery and the amount used for the evaluation of this parameter, LOD, LOQ and the way those limits were determined in the compared studies as well as in this study are given in Table S2 of the ESM.

The advantage of our method is the applicability to biological samples with low concentrations of OAs as we achieved very low detection and quantification limits while maintaining high recoveries. Solid-phase extraction of OAs is mainly used for food analysis and clinical chemistry where acid concentrations are considerably higher than in microorganisms, hence making existing methods unsuitable for the application to samples with low concentrations. Where stated, all studies used either the signal-to-noise ratio or the ratio of the regression standard deviation to the slope of the calibration to evaluate detection and quantification limits. Even though the calculation according to DIN 32645 results in higher detection and quantification limits than the estimation based on the SNR, our determined limits for succinic acid were still below the results of all studies evaluated. Aside from one exception for lactic acid, this holds true for all OAs analysed in any of the other studies. Therefore, our method is suitable to remove matrix interferences of microbiological samples and thereby enhances the sensitivity for the analysis of OAs.

### Application to *E. coli* and *P. minimum* cell pellets

Table 2 shows the concentrations determined in *E. coli* and *P. minimum* cells after bead beating using the newly developed method. Chromatograms of the measurements can be viewed in Fig. S5 of the ESM.

**Table 2:** Concentrations of selected OAs in *E. coli* and *P. minimum* cell pellet extracts

Organic acid	*E. coli*	*P. minimum*
	(μg/g_wet cell weight_ ± confidence range)	
Pyruvic acid	32 ± 10	nd
Benzoic acid	3.4 ± 0.6	2.7 ± 0.57
Succinic acid	622 ± 4.5	9.9 ± 0.59
Fumaric acid	22 ± 2.0	2.0 ± 1.9
Malic acid	58 ± 10	< LOD
Citric acid	13 ± 14	806 ± 144
Isocitric acid	nd	6.3 ± 9.9
Malonic acid	nd	< LOD
α-Ketoglutaric acid	72 ± 26	34 ± 25

Concentrations are given as mean of three biological replicates measured three times each

*nd* not detected

As some of the compounds selected for this study are linked to specific metabolic pathways which are not expected to occur in the main metabolism of the organisms chosen to apply this method to as a proof of concept, the number of identified and quantified acids is limited. It needs to be considered that recoveries of individual acids are not included into the calculations for the concentrations. Therefore, actual concentrations may be higher for pyruvic and malonic acid and lower for malic, citric, isocitric and α-ketoglutaric acid. However, we could show that the method developed in this study is suitable for prokaryotic and eukaryotic microorganisms and can be used to investigate and distinguish differences in the respective metabolomes. Contaminations for the acids quantified in cell extracts were excluded by analysing 15 water and 7 SPE blank samples over the course of the study (ESM, Table S6).

## Conclusion

We developed a method for the selective extraction, analysis and quantification of OAs out of aqueous phases based on anionic solid-phase extraction followed by GC-MS measurement. This method may be extended to higher molecular weight acids and is not limited to cell extracts. Additionally, it may be used to examine alterations in the metabolism in response to growth phases or changes in the substrate supplied to the microorganism as well as for observations in other sample sets like culture media or natural samples. Method validation was performed to show the stability of the method. We could demonstrate that the method is especially useful for biological samples with low concentrations of the target compounds. Due to the high range of polarity within the complex mixture of compounds chosen for this study, overall satisfying results could be achieved when considering problems arising for certain acids with additional functional groups. Finally, the method was successfully applied to bead beater extracts from *E. coli* and *P. minimum*.

## Electronic supplementary material

ESM 1 (DOCX 5.31 mb)

## Data Availability

The datasets generated and analysed during the current study are available from the corresponding author on reasonable request.
