# Supplementary material for: Method development and validation for the quantification of organic acids in microbial samples using anionic exchange solid-phase extraction and gas chromatography-mass spectrometry
Source: Anal Bioanal Chem. 2020 Sep 24;412(27):7491–503. doi: 10.1007/s00216-020-02883-3 (PMC7533261; doi:10.1007/s00216-020-02883-3)
Supplement: Supplementary file 1 — (DOCX 5.31 mb) [file 216_2020_2883_MOESM1_ESM.docx]

Electronic Supplementary Material

**Method development and validation for the quantification of organic acids in microbial samples using anionic exchange solid-phase extraction and gas chromatography-mass spectrometry**

Simone Heyen^1^, Barbara M. Scholz-Böttcher^1^, Ralf Rabus^1^, Heinz Wilkes^1*^

^1^ Institute for Chemistry and Biology of the Marine Environment (ICBM), Carl von Ossietzky University of Oldenburg, P.O. Box 2503, 26111 Oldenburg, Germany

^*^Corresponding Author: heinz.wilkes@uni-oldenburg.de

Analytical and Bioanalytical Chemistry

Supplement content: 18 pages (cover page included), 6 tables, 5 figures, 1 text passage, references

**Table S1** Workup procedures used for the different SPE cartridges tested

|  | Cartridge | | | | | | |
| --- | --- | --- | --- | --- | --- | --- | --- |
| Property | Phenomenex Strata X-AW | Supelclean LC-SAX | Chromabond HR-XA | Chromabond HR-XA | Chromabond PS-OH^−^ | Chromabond SB | Waters Oasis MAX |
| Bed size | 3 mL, 60 mg | 3 mL, 60 mg | 3 mL, 60 mg | 3 mL, 200 mg | 3 mL, 200 mg | 3 mL, 500 mg | 6 mL, 150 mg |
| Conditioning | 2 mL MeOH  + 2 mL H_2_O | 2 mL MeOH  + 2 mL 5% NH_4_OH | 2 mL MeOH  + 2 mL H_2_O | 5 mL MeOH  + 5 mL H_2_O | 3 mL MeOH  + 3 mL H_2_O | 3 mL MeOH  + 3 mL H_2_O | 4 mL MeOH  + 4 mL H_2_O |
| Sample | Basic, 5% NH_4_OH used (pH 9) | Basic, 5% NH_4_OH used (pH 9) | Basic, 0.1 M NaOH used (pH 9) | Basic, 0.1 M NaOH used (pH 9) | Basic, 5% NH_4_OH used (pH 9) | Basic, 5% NH_4_OH used (pH 9) | Basic, 5% NH_4_OH used (pH 9) |
| Wash | 2 mL 25 mM ammonium acetate  + 2 mL MeOH | 4 mL 5% NH_4_OH | 1 mL 0.1 M NaOH | 2 mL 0.1 M NaOH | 3 mL 5% NH_3_  + 3 mL MeOH/H_2_0 (20:80, v/v) | 3 mL 5% NH_3_ + 3 mL MeOH/H_2_0 (20:80, v/v) | 4 mL 5% NH_4_OH + 4 mL MeOH |
| Drying | under vacuum | under vacuum | with N_2_ | with N_2_ | / | / | / |
| Elution | 2 x 1 mL  5% FA in MeOH | 2 x 1 mL  5% FA in MeOH | 2 x 1 mL 5% FA in MeOH | 2 x 2 mL  5% FA in MeOH | 2 x 2 mL MeOH/Acetone  (1:1, v/v) + 5% AcOH | 2 x 2 mL  MeOH/Acetone  (1:1, v/v) + 5% AcOH | 2 x 2 mL  5% FA in MeOH |

AcOH. acetic acid; FA. formic acid

**
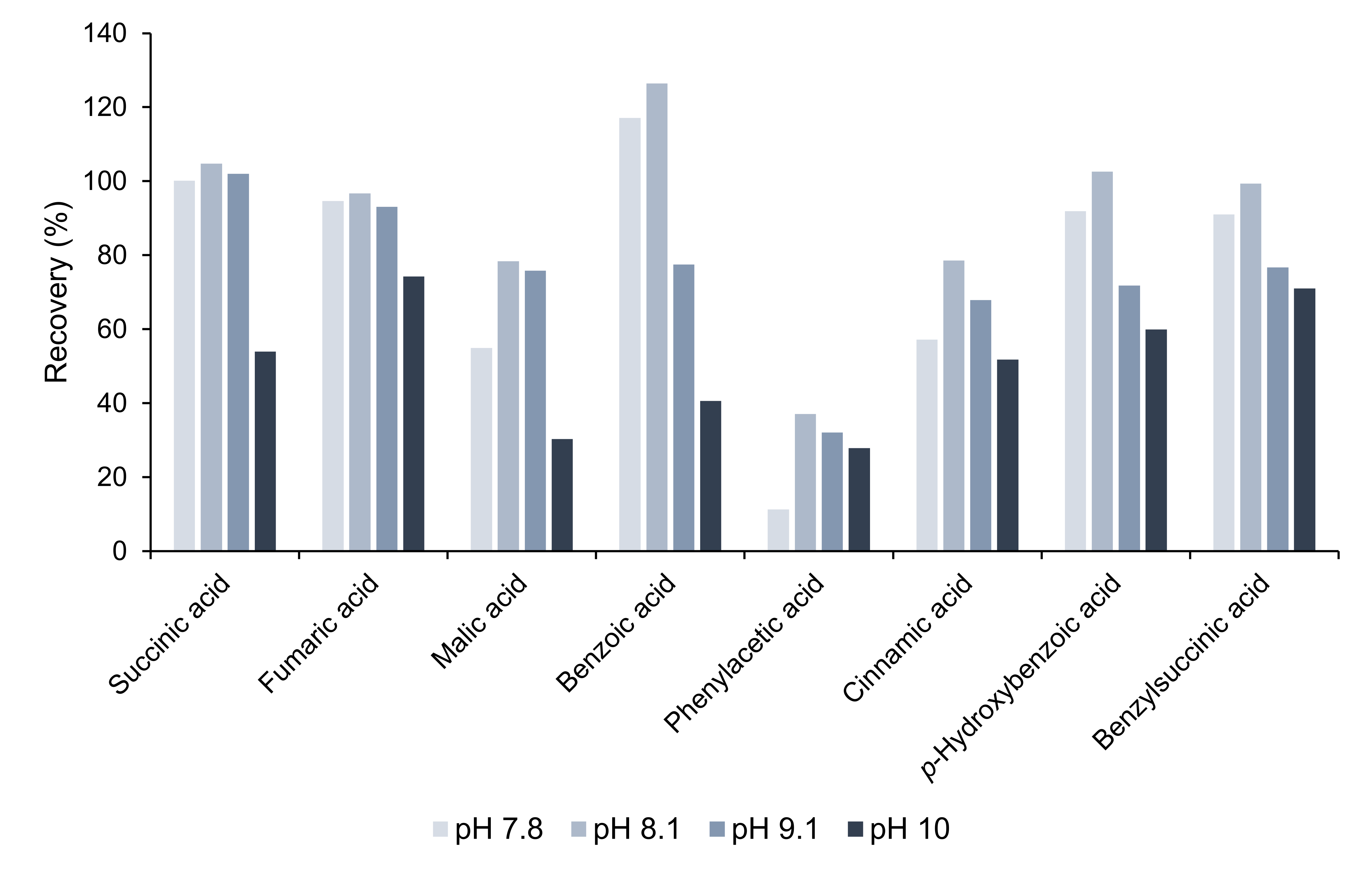
**

**Fig. S1** Recoveries of selected low molecular weight organic acids depending on of the pH value of the sample. Recoveries differ from those given in the main text, as these experiments were performed at one concentration and with a different method than applied for the validation


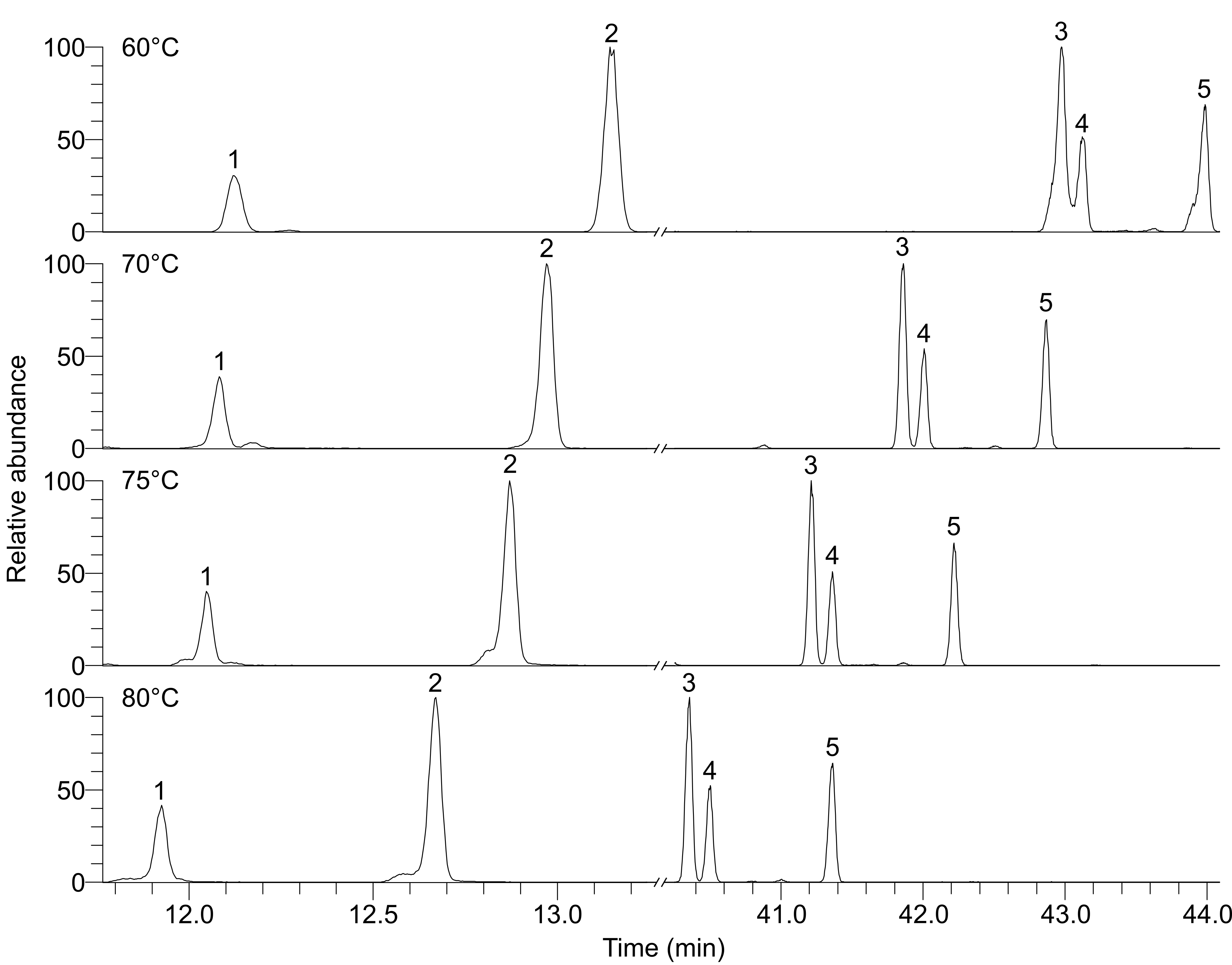


**Fig. S2** Effect of different starting temperatures of the GC-MS program on peak shapes. 1. Lactic acid; 2. pyruvic acid; 3. citric acid; 4. isocitric acid; 5. benzylsuccinic acid. Mass traces for the first part (retention time 11.77 – 13.24 min). *m/z* 189, 191, 217 and 219; mass traces used in the second part (40.25 – 44.09 min). *m/z* 175, 190, 262, 293, 321, 337, 352, 375 and 465. Retention times were adjusted to simplify the display


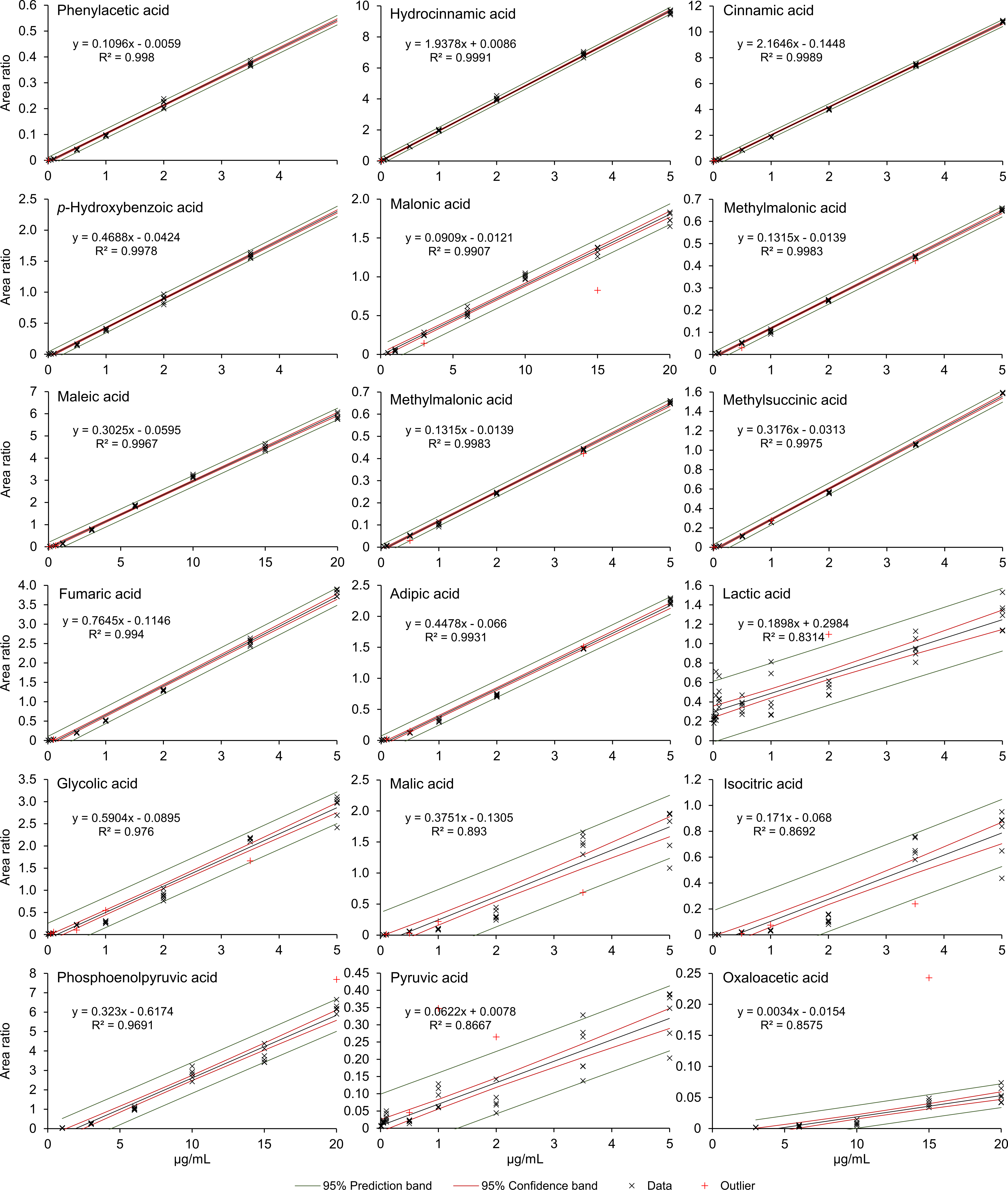


**Fig. S3** Calibration parameters for the organic acids not depicted in the main text. Each graph shows area ratios of the acids with the corresponding internal standard for all measured concentrations and replications. Outliers eliminated via the Grubbs test are marked with a red cross. The regression lines in black including the formula and the squared correlation coefficients are displayed as well as the 95% confidence (red) and prediction (green) bands


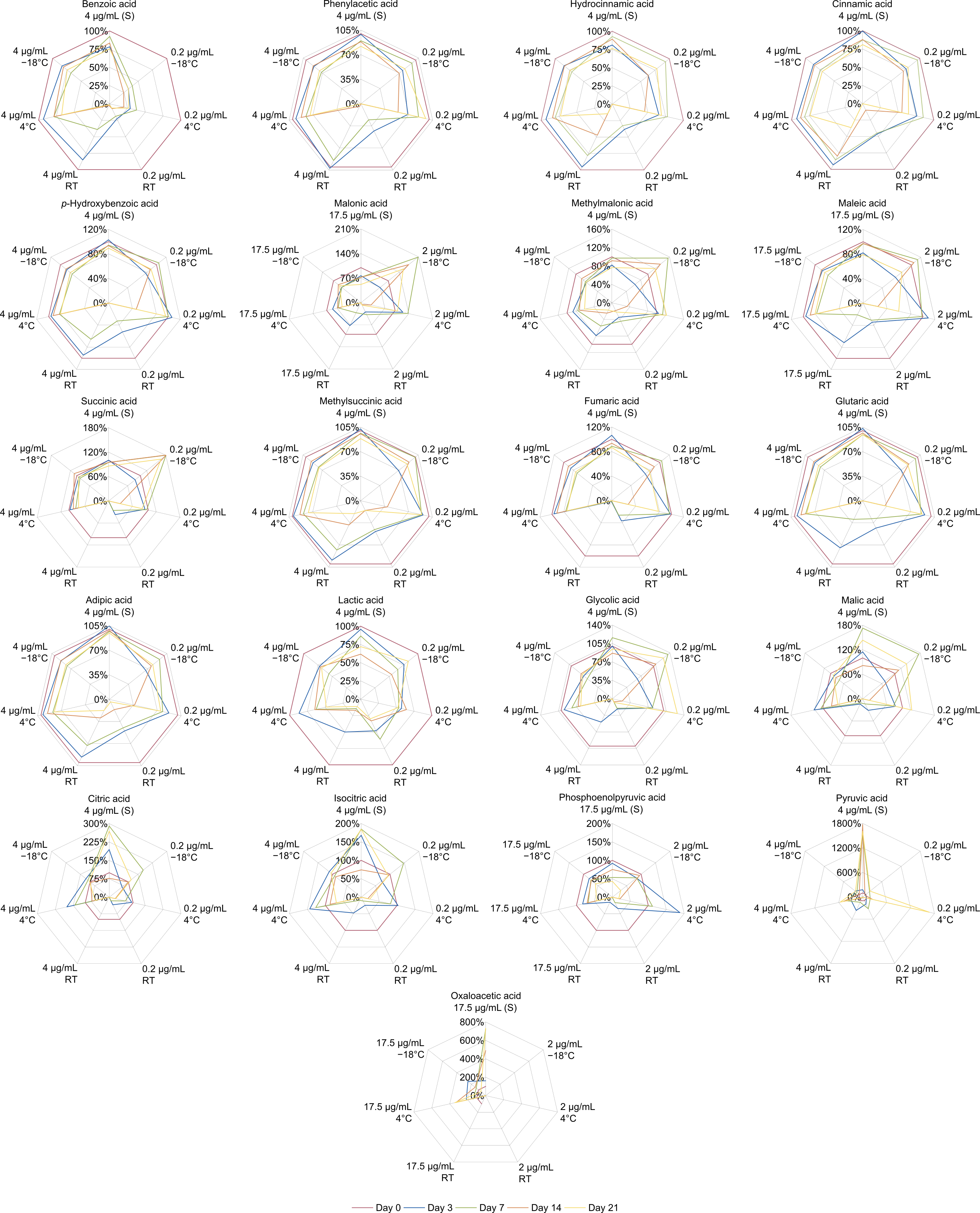


**Fig. S4** Storage stability over 21 days displayed as net graphs of the OAs not depicted in the main text. Mean values of relative intensities measured on each day are given as percentage of Day 0. (S) Diluted stock solution stored at −18°

**Table S2** Recovery, limit of detection and quantification of organic acids determined in different studies applying solid-phase extraction

|  | Matrix | Analytes | Technique | SPE material | Derivatization | Recovery (%) | Amount used for recovery (µg on cartridge) | LOD (ng on column) | LOQ (ng on column) | Determination LOD/LOQ | Reference |
| --- | --- | --- | --- | --- | --- | --- | --- | --- | --- | --- | --- |
|  | *E. coli* and *P.minimum* cells | 24 OAs | SPE GC-MS | Waters Oasis MAX | MSTFA |  |  |  |  | DIN 32645 | This study |
|  |  | Lactic acid |  |  |  | 27 | 4 - 10 | 0.115 | 1.367 |  |  |
|  |  | Glycolic acid |  |  |  | 5.5 | 0.02 - 10 | 0.041 | 0.175 |  |  |
|  |  | Pyruvic acid |  |  |  | 19 | 0.02 - 10 | 0.05 | 0.255 |  |  |
|  |  | Methylmalonic acid |  |  |  | 27 | 0.2 - 10 | 0.021 | 0.072 |  |  |
|  |  | Benzoic acid |  |  |  | 100 | 0.02 - 10 | 0.03 | 0.11 |  |  |
|  |  | Phenylacetic acid |  |  |  | 105 | 0.1 - 10 | 0.015 | 0.005 |  |  |
|  |  | Succinic acid |  |  |  | 109 | 0.02 - 10 | 0.022 | 0.075 |  |  |
|  |  | Methylsuccinic acid |  |  |  | 103 | 0.02 - 10 | 0.019 | 0.062 |  |  |
|  |  | Fumaric acid |  |  |  | 100 | 0.1 - 10 | 0.003 | 0.005 |  |  |
|  |  | Glutaric acid |  |  |  | 104 | 0.02 - 10 | 0.015 | 0.053 |  |  |
|  |  | Hydrocinnamic acid |  |  |  | 100 | 0.02 - 10 | 0.008 | 0.025 |  |  |
|  |  | Malic acid |  |  |  | 151 | 0.1 - 10 | 0.098 | 1.224 |  |  |
|  |  | Adipic acid |  |  |  | 104 | 0.02 - 10 | 0.03 | 0.11 |  |  |
|  |  | Cinnamic acid |  |  |  | 100 | 0.1 - 10 | 0.004 | 0.015 |  |  |
|  |  | Pimelic acid |  |  |  | 101 | 0.02 - 10 | 0.023 | 0.105 |  |  |
|  |  | *p*-Hydroxybenzoic acid |  |  |  | 111 | 0.02 - 10 | 0.033 | 0.149 |  |  |
|  |  | Citric acid |  |  |  | 125 | 0.2 - 10 | 0.045 | 0.172 |  |  |
|  |  | Isocitric acid |  |  |  | 161 | 0.2 - 10 | 0.038 | 0.121 |  |  |
|  |  | Benzylsuccinic acid |  |  |  | 110 | 0.1 - 10 | 0.074 | 0.594 |  |  |
|  |  | Malonic acid |  |  |  | 54 | 20 - 40 | 0.121 | 0.344 |  |  |
|  |  | Maleic acid |  |  |  | 0.3 | 20 - 40 | 0.272 | 0.861 |  |  |
|  |  | Phosphoenolpyruvic acid |  |  |  | 0 | na | 1.477 | 5.899 |  |  |
|  |  | α-Ketoglutaric acid |  |  |  | 273 | 2 - 40 | 0.437 | 1.594 |  |  |
|  |  | Oxaloacetic acid |  |  |  | 16 | 20 - 40 | 5.577 | 20.564 |  |  |

**Table S2** Continued

|  | Matrix | Analytes | Technique | SPE material | Derivatisation | Recovery (%) | Amount used for recovery (µg on cartridge) | LOD (ng on column) | LOQ (ng on column) | Determination LOD/LOQ | Reference |
| --- | --- | --- | --- | --- | --- | --- | --- | --- | --- | --- | --- |
|  | Honey | 5 OAs | SPE HPLC | Waters Accell Plus QMA | / |  |  |  |  |  | Suárez-Luque et al. (2002) |
|  |  | Malic acid |  |  |  | 102 | na |  |  |  |  |
|  |  | Maleic acid |  |  |  | 103 | na |  |  |  |  |
|  |  | Citric acid |  |  |  | 101 | na |  |  |  |  |
|  |  | Succinic acid |  |  |  | 99 | na |  |  |  |  |
|  |  | Fumaric acid |  |  |  | 103 | na |  |  |  |  |
|  |  |  |  |  |  |  |  |  |  |  |  |
|  | Wine | Polyphenols, sugars and OAs | SPE HPLC | Strata SDB-L | / |  |  |  |  | SNR 3 / 10 | Villiers et al. (2004) |
|  |  | Citric acid |  |  |  | 96 | 1000 | 15 | 51 |  |  |
|  |  | Malic acid |  |  |  | 98 | 2000 | 22 | 75 |  |  |
|  |  | Succinic acid |  |  |  | 81 | 1250 | 37 | 12.5 |  |  |
|  |  | Lactic acid |  |  |  | 75 | 1350 | 37 | 12.2 |  |  |
|  |  |  |  |  |  |  |  |  |  |  |  |
|  | Wine | 6 OAs | SPE HPLC | Supelclean LC-18 | / |  |  |  |  | SNR 3 / 10 | Tašev et al. (2016) |
|  |  | Malic acid |  |  |  | 99 / 105 | 700 / 1030 | 118 | 389 |  |  |
|  |  | Lactic acid |  |  |  | 96 / 97 | 345 / 491.5 | 136 | 449 |  |  |
|  |  | Citric acid |  |  |  | 105 / 96 | 170 / 266 | 128 | 423 |  |  |
|  |  | Succinic acid |  |  |  | 104 / 96 | 540 / 681.5 | 123 | 406 |  |  |

**Table S2** Continued

|  | Matrix | Analytes | Technique | SPE material | Derivatization | Recovery (%) | Amount used for recovery (µg on cartridge) | LOD (ng on column) | LOQ (ng on column) | Determination LOD/LOQ | Reference |
| --- | --- | --- | --- | --- | --- | --- | --- | --- | --- | --- | --- |
|  | Honey | 29 OAs | SPE HPLC | Bond-Elut SAX | / |  |  |  |  | na | Cherchi et al. (1994) |
|  |  | Citric acid |  |  |  | 100 / 97 / 99 | 250 / 500 / 1000 | 10 |  |  |  |
|  |  | Fumaric acid |  |  |  | 99 / 98 / 99 | 1 / 2.5 / 5 | 0.04 |  |  |  |
|  |  | Glutaric acid |  |  |  | 100 / 99 / 101 | 500 / 1000 / 2000 | 10 |  |  |  |
|  |  | Glycolic acid |  |  |  | 99 / 97 / 99 | 500 / 1000 / 2000 | 10 |  |  |  |
|  |  | Lactic acid |  |  |  | 99 / 94 / 96 | 250 / 500 / 1000 | 20 |  |  |  |
|  |  | α-Ketoglutaric acid |  |  |  | 98 / 98 / 98 | 50 / 100 / 250 | 4 |  |  |  |
|  |  | Malic acid |  |  |  | 101 / 102 / 100 | 250 / 500 / 1000 | 10 |  |  |  |
|  |  | Malonic acid |  |  |  | 99 / 100 / 100 | 250 / 500 / 1000 | 4 |  |  |  |
|  |  | Methylmalonic acid |  |  |  | 99 / 97 / 96 | 250 / 500 / 1000 | 16 |  |  |  |
|  |  | Pyruvic acid |  |  |  | 102 / 97 / 100 | 50 / 100 / 250 | 10 |  |  |  |
|  |  | Succinic acid |  |  |  | 96 / 96 / 97 | 250 / 500 / 1000 | 7 |  |  |  |
|  |  | Isocitric acid |  |  |  | 95 / 98 / 90 | 500 / 1000 / 2000 | 23 |  |  |  |
|  |  |  |  |  |  |  |  |  |  |  |  |
|  | Non-small cell lung cancer | 6 OAs | SPE LC-MS/MS | Clean-up CUQAX extraction columns | / |  |  |  |  | SNR 3 / 10 | Klupczynska et al. (2016) |
|  |  | Fumaric acid |  |  |  | 96 / 94 / 96 | 0.2 / 0.5 / 3 | 0.046 | 0.104 |  |  |
|  |  | Glutaric acid |  |  |  | 114 / 107 / 111 | 0.2 / 0.5 / 3 | 0.04 | 0.106 |  |  |
|  |  | Lactic acid |  |  |  | 100 / 103 / 102 | 0 02 / 0.1 / 0.4 | < 0.0009 | < 0.0009 |  |  |
|  |  | Succinic acid |  |  |  | 98 / 104 / 101 | 0.2 / 0.5 / 3 | 0.047 | 0.094 |  |  |
|  |  |  |  |  |  |  |  |  |  |  |  |
|  | Urine | 25 OAs | SPE GC-MS | Sep-Pak Vac RC, Accell Plus QMA | MSTFA |  |  |  |  |  | Liu et al. (2004) |
|  |  | Citric acid |  |  |  | 105 | na | < 0.96 |  |  |  |
|  |  | Fumaric acid |  |  |  | 100 | na | < 0.58 |  |  |  |
|  |  | Glutaric acid |  |  |  | 100 | na | < 0.66 |  |  |  |
|  |  | Glycolic acid |  |  |  | 80 | na | < 0.38 |  |  |  |
|  |  | Lactic acid |  |  |  | 89 | na | < 0.45 |  |  |  |
|  |  | Methylsuccinic acid |  |  |  | 120 | na | < 0.66 |  |  |  |
|  |  | Methylmalonic acid |  |  |  | 52 | na | 0.59 - 1.48 |  |  |  |
|  |  | Succinic acid |  |  |  | 106 | na | < 0.59 |  |  |  |
|  |  | Malonic acid |  |  |  | na |  | > 52 |  |  |  |

**Table S2** Continued

|  | Matrix | Analytes | Technique | SPE material | Derivatization | Recovery (%) | Amount used for recovery (µg on cartridge) | LOD (ng on column) | LOQ (ng on column) | Determination LOD/LOQ | Reference |
| --- | --- | --- | --- | --- | --- | --- | --- | --- | --- | --- | --- |
|  | Coffee | 7 OAs | SPE HPLC | Strata SAX | / |  |  |  |  | 3σ/S / 10σ/S | Rodrigues et al. (2007) |
|  |  | Citric acid |  |  |  | 122 | na | 400 | 1360 |  |  |
|  |  | Malic acid |  |  |  | 59 | na | 8 | 20 |  |  |
|  |  | Pyruvic acid |  |  |  | 88 | na | 120 | 620 |  |  |
|  |  | Succinic acid |  |  |  | 97 | na | 600 | 2020 |  |  |
|  |  |  |  |  |  |  |  |  |  |  |  |
|  | Urine | 58 OAs | SPE GC | Diethylaminoehyl-Sephadex A-25 anion exchange | BSTFA |  |  |  |  |  | Verhaeghe et al. (1988) |
|  |  | Lactic acid |  |  |  | 61 | 70 |  |  |  |  |
|  |  | Glycolic acid |  |  |  | 72 | 70 |  |  |  |  |
|  |  | Pyruvic acid |  |  |  | 60 | 70 |  |  |  |  |
|  |  | α-Ketoglutaric acid |  |  |  | 91 | 70 |  |  |  |  |
|  |  | Malonic acid |  |  |  | 117 | 70 |  |  |  |  |
|  |  | Succinic acid |  |  |  | 79 | 70 |  |  |  |  |
|  |  | Fumaric acid |  |  |  | 107 | 70 |  |  |  |  |
|  |  | Methylmalonic acid |  |  |  | 98 | 70 |  |  |  |  |
|  |  | Glutaric acid |  |  |  | 72 | 70 |  |  |  |  |
|  |  | Adipic acid |  |  |  | 74 | 70 |  |  |  |  |
|  |  | Pimelic acid |  |  |  | 78 | 70 |  |  |  |  |
|  |  | Malic acid |  |  |  | 89 | 70 |  |  |  |  |
|  |  | Citric acid |  |  |  | 62 | 70 |  |  |  |  |
|  |  | Benzoic acid |  |  |  | 34 | 70 |  |  |  |  |
|  |  | Phenylacetic acid |  |  |  | 89 | 70 |  |  |  |  |

**Table S2** Continued

|  | Matrix | Analytes | Technique | SPE material | Derivatization | Recovery (%) | Amount used for recovery (µg on cartridge) | LOD (ng on column) | LOQ (ng on column) | Determination LOD/LOQ | Reference |
| --- | --- | --- | --- | --- | --- | --- | --- | --- | --- | --- | --- |
|  | Fruit juices | 8 OAs and 3 sugars | SPE HPLC | Varian Bond Elut SAX | / |  |  |  |  | SNR 3 | Chinnici et al. (2005) |
|  |  | Citric acid |  |  |  | 97 | na | 66 |  |  |  |
|  |  | Malic acid |  |  |  | 98 | na | 36 |  |  |  |
|  |  | Succinic acid |  |  |  | 98 | na | 102 |  |  |  |
|  |  | Fumaric acid |  |  |  | 99 | na | 15 |  |  |  |
|  |  |  |  |  |  |  |  |  |  |  |  |
|  | Wine | 7 OAs | SPE HPLC | Varian Bond Elut SAX |  |  |  |  |  | SNR 2 / 10 | Zotou et al. (2004) |
|  |  | Malic acid |  |  |  | 90 / 92 | 183.5 / 233.5 | 250 | 1000 |  |  |
|  |  | Lactic acid |  |  |  | 99 / 91 | 145 / 195 | 300 | 600 |  |  |
|  |  | Citric acid |  |  |  | 78 / 89 | 38 / 68 | 100 | 200 |  |  |
|  |  | Succinic acid |  |  |  | 86 / 81 | 373.5 / 473.5 | 1000 | 2000 |  |  |

na. not available; SNR. signal-to-noise ratio; σ/S. slope of the regression line divided by the standard deviation of the detector response; only overlapping OAs are listed

**Further statistical evaluation**

The Shapiro-Wilk and the David tests for Gaussian distribution, which are both suitable for small sample sets, resulted in contrary statements. At levels where the Shapiro-Wilk test gave a negative output, the values fell into the control range of the David test, and vice versa (Table S3). Only in one case both tests were negative. Overall, the Shapiro-Wilk test had seven rejected results, while the David test showed 35 out of 181 values being outside of the test ranges. Even when changing the confidence level of the David test to 95%, 15 concentration levels remained beyond the test ranges. Although the test for homogeneity of variances as described in DIN 38402 part 51 requires at least ten repetitions and the calibration had only six, we nevertheless tried to apply it [11]. However, it failed for all compounds (Table S4). When used with the results gained for the stability of the derivatized samples, where 15 values were available, the test had a positive feedback for lactic acid only. A second test to evaluate variances is Cochran’s C-test, which compares standard deviations across the complete calibration range. This test asks for a Gaussian distribution, which was not valid for all calibration levels, as the Shapiro-Wilk and the David tests showed. Secondly, degrees of freedom should be equal for all concentrations, otherwise it can be applied very conservatively by choosing the critical value given by the higher degree of freedom. When neglecting Gaussian distribution, results of Cochran’s C-test were positive for five out of 24 compounds (Table S4). The Mandel test for linearity failed for 16 out of 24 acids (Table S5). 13 of those calibrations had squared regression coefficients above 0.99 and two above 0.98. α-Ketoglutaric acid was the only compound with a lower R² of 0.94, which was still better than the R² of some other acids where the test did not react. In conclusion, the test encountered problems for the acids with the best fits in the linear range. Normally, when the Mandel test has a negative output, the calibration range needs to be narrowed down until the test value of the Mandel test is below the tabular value. In this case three calibration levels had to be omitted for most acids to fit the criteria of the test. This would result in very low calibration ranges and R² which are worse than the initial correlations given for the complete calibration range. Therefore, it is not recommended to apply this test for calibration with a very good linear fit.

Different tests applied for the same purpose can give contrary results, as is shown here for the Shapiro-Wilk and the David tests. Considering the limited number of repetitions (n=6) a final statement about the presence or absence of a Gaussian distribution cannot be made. According to the test for homogeneity of variances, validation would not be possible because homogeneity is not given for all acids. Trying to optimise the data until all criteria are fulfilled would drastically limit method development and the applicability of these methods. Statistical evaluation should fit to the intended purpose of the developed method. When exact concentrations are needed, method validation should necessarily include such tests. However, in most cases trends in concentrations are of more interest than exact values when various factors, for example microorganisms, growth phases or substrates are compared, reducing the need for an in-depth statistical evaluation prior to the application when general validation criteria are met.

**Table S3**: Test values of the David test and output of the Shapiro-Wilk test. Values outside of the ranges for the David test or above the test value for the Shapiro-Wilk test are highlighted in grey

|  | David | | | | | | | | Shapiro-Wilk | | | | | | | |
| --- | --- | --- | --- | --- | --- | --- | --- | --- | --- | --- | --- | --- | --- | --- | --- | --- |
|  | µg/mL | | | | | | | | | | | | | | | |
|  | 0.01 | 0.05 | 0.1 | 0.5 | 1 | 2 | 3.5 | 5 | 0.01 | 0.05 | 0.1 | 0.5 | 1 | 2 | 3.5 | 5 |
| Benzoic acid | 2.474 | 2.799 | 2.641 | 2.855 | 2.777 | 2.193 | 3.050 | 2.371 | OK | OK | OK | OK | OK | OK | OK | OK |
| Phenylacetic acid | 2.679 | 2.552 | 2.688 | 2.416 | 2.965 | 2.571 | 2.663 | 2.485 | OK | OK | OK | OK | OK | OK | OK | OK |
| Hydrocinnamic acid | 2.323 | 2.589 | 2.711 | 2.232 | 2.430 | 2.961 | 2.948 | 2.528 | OK | OK | OK | OK | OK | OK | OK | OK |
| Cinnamic acid | 2.393 | 2.389 | 2.976 | 2.518 | 2.295 | 2.978 | 2.524 | 2.665 | OK | OK | OK | OK | OK | OK | OK | OK |
| *p*-Hydroxybenzoic acid | 2.628 | 3.071 | 2.349 | 2.755 | 2.409 | 2.727 | 2.588 | 2.868 | OK | OK | OK | OK | OK | OK | OK | OK |
| Benzylsuccinic acid | 2.173 | 2.602 | 2.832 | 2.920 | 2.661 | 2.776 | 2.938 | 2.802 | OK | OK | OK | OK | OK | OK | OK | OK |
| Methylmalonic acid | na | 2.832 | 2.919 | 2.482 | 2.999 | 2.302 | 2.319 | 2.420 | nA | OK | OK | OK | OK | OK | OK | OK |
| Succinic acid | 2.890 | 2.637 | 2.334 | 2.356 | 2.757 | 2.499 | 2.112 | 2.681 | OK | OK | OK | OK | OK | OK | OK | OK |
| Methylsuccinic acid | 2.574 | 2.770 | 2.673 | 2.429 | 2.420 | 2.845 | 2.543 | 2.587 | OK | OK | OK | OK | OK | OK | OK | OK |
| Fumaric acid | 2.872 | 2.311 | 2.354 | 2.524 | 3.115 | 2.644 | 2.695 | 2.385 | OK | OK | OK | OK | OK | OK | OK | OK |
| Glutaric acid | 2.838 | 2.394 | 2.483 | 2.602 | 2.008 | 2.482 | 2.610 | 2.346 | OK | OK | OK | OK | OK | OK | OK | !!! |
| Adipic acid | 2.771 | 2.754 | 2.332 | 2.424 | 2.700 | 2.777 | 2.679 | 2.567 | OK | OK | OK | OK | OK | OK | OK | OK |
| Pimelic acid | 2.607 | 2.977 | 2.570 | 2.369 | 2.696 | 2.565 | 2.539 | 2.300 | OK | OK | OK | !!! | OK | OK | OK | OK |
| Lactic acid | 2.655 | 2.603 | 2.845 | 2.805 | 2.363 | 2.274 | 2.820 | 2.643 | OK | OK | OK | OK | OK | OK | OK | OK |
| Glycolic acid | 2.860 | 2.684 | 2.575 | 2.156 | 2.499 | 2.892 | 2.477 | 2.613 | OK | OK | OK | OK | OK | OK | OK | OK |
| Malic acid | na | 2.992 | 2.563 | 2.479 | 2.538 | 2.591 | 2.607 | 2.423 | nA | OK | OK | OK | OK | OK | OK | !!! |
| Citric acid | na | 2.755 | 2.357 | 2.123 | 2.390 | 2.461 | 2.468 | 2.752 | nA | OK | !!! | OK | OK | OK | OK | OK |
| Isocitric acid | na | 2.548 | 2.985 | 2.084 | 2.667 | 2.485 | 2.293 | 2.641 | nA | OK | OK | OK | OK | OK | OK | OK |
| Pyruvic acid | 2.498 | 2.703 | 2.588 | 2.617 | 2.231 | 2.666 | 2.608 | 2.467 | OK | OK | OK | OK | OK | OK | OK | OK |
|  | µg/mL | | | | | | | | | | | | | | | |
|  | 0.1 | 0.5 | 1 | 3 | 6 | 10 | 15 | 20 | 0.1 | 0.5 | 1 | 3 | 6 | 10 | 15 | 20 |
| Malonic acid | na | 2.780 | 2.892 | 2.548 | 2.632 | 2.623 | 2.297 | 2.466 | na | OK | OK | !!! | OK | OK | OK | OK |
| Maleic acid | 2.687 | 2.698 | 2.481 | 3.105 | 2.984 | 2.892 | 2.785 | 2.501 | OK | OK | OK | OK | OK | OK | OK | OK |
| Phosphoenolpyruvic acid | na | na | 2.367 | 2.809 | 2.765 | 2.941 | 2.420 | 2.731 | na | na | OK | OK | OK | OK | OK | OK |
| α-Ketoglutaric acid | na | 2.751 | 3.012 | 1.909 | 2.430 | 2.885 | 2.349 | 2.788 | na | OK | OK | !!! | OK | OK | OK | OK |
| Oxaloacetic acid | na | na | na | 2.451 | 2.520 | 2.942 | 2.522 | 2.544 | na | na | na | !!! | OK | OK | OK | OK |

na. not available

**Table S4**: Test for the homogeneity of variances for the calibration and for the stability of the derivatized samples as well as results of the Cochran test applied to the calibration. Values below the tabulated value and therefore within the permitted range a highlighted in grey

|  | Homogeneity of Variances | | | | Cochran | |
| --- | --- | --- | --- | --- | --- | --- |
|  | Calibration | | Stability of derivatized samples | | Calibration | |
|  | Test Value | Tabular Value | Test Value | Tabular Value | Test Value | Tabular Value |
| Benzoic acid | 15.3 | 15.5 | 11 | 3.698 | 0.432 | 0.447 |
| Phenylacetic acid | 31464 | 15.5 | 722 | 3.698 | 0.569 | 0.447 |
| Hydrocinnamic acid | 2105 | 15.5 | 208 | 3.698 | 0.401 | 0.447 |
| Cinnamic acid | 863 | 15.5 | 160 | 3.698 | 0.531 | 0.447 |
| *p*-Hydroxybenzoic acid | 3144 | 11.0 | 1713 | 3.698 | 0.536 | 0.447 |
| Benzylsuccinic acid | 14027 | 11.0 | 5999 | 3.698 | 0.495 | 0.447 |
| Malonic acid | 1313 | 11.0 | 94 | 3.698 | 0.461 | 0.447 |
| Methylmalonic acid | 761 | 11.0 | 119 | 3.698 | 0.463 | 0.447 |
| Maleic acid | 32827 | 15.5 | 104 | 3.698 | 0.478 | 0.447 |
| Succinic acid | 176 | 11.0 | 125 | 3.698 | 0.768 | 0.447 |
| Methylsuccinic acid | 3080 | 15.5 | 267 | 3.698 | 0.353 | 0.447 |
| Fumaric acid | 63664 | 11.0 | 1609 | 3.698 | 0.485 | 0.447 |
| Glutaric acid | 395 | 11.4 | 630 | 3.698 | 0.610 | 0.447 |
| Adipic acid | 2121 | 11.0 | 864 | 3.698 | 0.629 | 0.447 |
| Pimelic acid | 101105 | 15.5 | 1823 | 3.698 | 0.805 | 0.447 |
| Lactic acid | 33 | 11.0 | 1.13 | 3.698 | 0.364 | 0.447 |
| Glycolic acid | 8909 | 11.0 | 247 | 3.698 | 0.866 | 0.447 |
| Malic acid | 273305 | 11.0 | 3881 | 3.698 | 0.842 | 0.447 |
| Citric acid | 23029414 | 15.5 | 8183 | 3.698 | 0.889 | 0.447 |
| Isocitric acid | 3674982 | 15.5 | 6605 | 3.698 | 0.843 | 0.447 |
| Phosphoenolpyruvic acid | 689 | 11.4 | 5487 | 3.698 | 0.509 | 0.447 |
| Pyruvic acid | 5979 | 11.0 | 53 | 3.698 | 0.421 | 0.447 |
| α-Ketoglutaric acid | 26942 | 11.4 | 38 | 3.698 | 0.450 | 0.447 |
| Oxaloacetic acid | 1905 | 15.5 | na | na | 0.767 | 0.447 |

na. not available

**Table S5** Results of the Mandel test including R² of the respective regression. Squared correlation coefficients of the organic acids for which the Mandel test declined the calibration are highlighted in grey

|  | Test Value | Tabular Value | Output | R² |
| --- | --- | --- | --- | --- |
| Benzoic acid | 21.93 | 7.28 | !!!! | 0.9994 |
| Phenylacetic acid | 1.28 | 7.25 | OK | 0.9980 |
| Hydrocinnamic acid | 17.29 | 7.25 | !!!! | 0.9991 |
| Cinnamic acid | 30.09 | 7.25 | !!!! | 0.9989 |
| *p*-Hydroxybenzoic acid | 6.14 | 7.23 | OK | 0.9978 |
| Benzylsuccinic acid | 58.89 | 7.23 | !!!! | 0.9869 |
| Malonic acid | 9.98 | 7.37 | !!!! | 0.9907 |
| Methylmalonic acid | 28.75 | 7.37 | !!!! | 0.9983 |
| Maleic acid | 12.73 | 7.26 | !!!! | 0.9967 |
| Succinic acid | 41.06 | 7.25 | !!!! | 0.9994 |
| Methylsuccinic acid | 112.23 | 7.26 | !!!! | 0.9975 |
| Fumaric acid | 34.73 | 7.25 | !!!! | 0.9940 |
| Glutaric acid | 82.77 | 7.31 | !!!! | 0.9950 |
| Adipic acid | 63.35 | 7.28 | !!!! | 0.9931 |
| Pimelic acid | 76.88 | 7.30 | !!!! | 0.9874 |
| Lactic acid | 2.79 | 7.25 | OK | 0.8314 |
| Glycolic acid | 4.10 | 7.31 | OK | 0.9760 |
| Malic acid | 1.58 | 7.42 | OK | 0.8930 |
| Citric acid | 0.96 | 7.44 | OK | 0.7799 |
| Isocitric acid | 1.98 | 7.42 | OK | 0.8692 |
| Phosphoenolpyruvic acid | 5.30 | 7.50 | OK | 0.9691 |
| Pyruvic acid | 0.94 | 7.28 | OK | 0.8667 |
| α-Ketoglutaric acid | 7.57 | 7.37 | !!!! | 0.9387 |
| Oxaloacetic acid | −1.59 | 7.77 | OK | 0.8575 |





**Fig. S5** Total ion chromatograms of **a** *E. coli* and **b** *P. minimum* cell pellets after bead beating and solid phase extraction. *tentatively identified metabolites based on mass spectra **compounds part of the growth medium

**Table S6** Calculated concentrations of OAs detected in water after derivatization (double distilled, pH 9) and after solid phase extraction performed with unspiked matrix

|  | Lactic Acid | Glycolic Acid | Pyruvic Acid | Benzoic Acid | Succinic Acid |
| --- | --- | --- | --- | --- | --- |
|  | µg/mL | | | | |
| Blank 1 | −1.13 | 0.11 | −0.24 | −0.09 | - |
| Blank 2 | −0.05 | 0.11 | −0.24 | −0.03 | - |
| Blank 3 | −0.17 | 0.11 | - | 0.02 | - |
| Blank 4 | −1.78 | 0.10 | −0.24 | −0.13 | - |
| Blank 5 | −1.48 | 0.09 | −0.22 | −0.09 | - |
| Blank 6 | −0.40 | 0.09 | −0.23 | −0.10 | - |
| Blank 7 | −1.39 | 0.09 | −0.24 | −0.11 | - |
| Blank 8 | −0.73 | 0.11 | −0.24 | −0.11 | - |
| Blank 9 | −0.94 | 0.11 | −0.24 | −0.11 | - |
| Blank 10 | −1.31 | 0.11 | −0.24 | −0.11 | - |
| Blank 11 | −1.43 | 0.09 | −0.25 | −0.11 | - |
| Blank 12 | −0.11 | 0.14 | −0.22 | −0.09 | - |
| Blank 13 | 1.69 | 0.09 | −0.24 | −0.14 | - |
| Blank 14 | −0.85 | 0.13 | −0.23 | −0.13 | - |
| Blank 15 | −1.35 | 0.11 | −0.22 | −0.09 | - |
| Blank SPE 1 | −1.79 | 0.09 | −0.25 | −0.14 | −0.06 |
| Blank SPE 2 | −1.79 | 0.10 | −0.25 | −0.13 | −0.06 |
| Blank SPE 3 | −1.33 | 0.12 | −0.25 | −0.13 | −0.06 |
| Blank SPE 4 | −1.05 | 0.11 | −0.24 | −0.12 | −0.06 |
| Blank SPE 5 | −1.78 | 0.11 | −0.25 | −0.12 | −0.06 |
| Blank SPE 6 | −1.57 | 0.10 | −0.23 | −0.11 | −0.06 |
| Blank SPE 7 | −1.43 | 0.11 | −0.21 | −0.07 | −0.05 |

**References**

1. Suárez-Luque S, Mato I, Huidobro JF, Simal-Lozano J. Solid-phase extraction procedure to remove organic acids from honey. J Chromatogr B. 2002;770:77–82

2. Villiers A de, Lynen F, Crouch A, Sandra P. Development of a solid-phase extraction procedure for the simultaneous determination of polyphenols, organic acids and sugars in wine. Chromatographia. 2004;59:403–409

3. Ivanova-Petropulos V, Tašev K, Stefova M. HPLC method validation and application for organic acid analysis in wine after solid-phase extraction. Maced J Chem Chem Eng. 2016;35:225

4. Cherchi A, Spanedda L, Tuberoso C, Cabras P. Solid-phase extraction and high-performance liquid chromatographic determination of organic acids in honey. J Chromatogr A. 1994;669:59–64

5. Klupczynska A, Plewa S, Dyszkiewicz W, Kasprzyk M, Sytek N, Kokot ZJ. Determination of low-molecular-weight organic acids in non-small cell lung cancer with a new liquid chromatography-tandem mass spectrometry method. J Pharm Biomed Anal. 2016;129:299–309

6. Liu A, Kushnir MM, Roberts WL, Pasquali M. Solid phase extraction procedure for urinary organic acid analysis by gas chromatography mass spectrometry. J Chromatogr B. 2004;806:283–287

7. Rodrigues CI, Marta L, Maia R, Miranda M, Ribeirinho M, Máguas C. Application of solid-phase extraction to brewed coffee caffeine and organic acid determination by UV/HPLC. J Food Compost Anal. 2007;20:440–448

8. Verhaeghe BJ, Lefevere MF, Leenheer AP de. Solid-phase extraction with strong anion-exchange columns for selective isolation and concentration of urinary organic acids. Clin Chem. 1988;34:1077–1083

9. Chinnici F, Spinabelli U, Riponi C, Amati A. Optimization of the determination of organic acids and sugars in fruit juices by ion-exclusion liquid chromatography. J Food Compost Anal. 2005;18:121–130

10. Zotou A, Loukou Z, Karava O. Method development for the determination of seven organic acids in wines by reversed-phase high performance liquid chromatography. Chromatographia. 2004;60:39–44

11. German Institute for Standardization. DIN 38402-51:2017-05, *German standard methods for the examination of water, waste water and sludge - General information (group A) - Part 51: Calibration of analytical methods - Linear calibration (A 51)*. Beuth Verlag, Berlin
